# Personality of Belgian physicians in a clinical leadership program

**DOI:** 10.1186/s12913-018-3645-1

**Published:** 2018-11-06

**Authors:** Neree Claes, Hannelore Storms, Valérie Brabanders

**Affiliations:** 1 Faculty of Medicine and Life Sciences, Hasselt University, Martelarenlaan 42, 3500 Hasselt, Belgium; 2 Faculty of Medicine and Life Sciences, Antwerp Management School, Sint-Jacobsmarkt 9-13, 2000 Antwerp, Belgium; 3 Wit-Gele Kruis Limburg vzw, Welzijnscampus 25, 3600 Genk, Belgium

**Keywords:** Leadership, Physicians, Healthcare, MBTI-assessment tool, Personality

## Abstract

**Background:**

Physician and non-physician leadership development programs aim to improve organizational performance. Although a significant, positive relation between physicians’ leadership skills and patient outcomes, staff satisfaction and staff retention has been found, physicians are not formally trained in clinical leadership skills during their physician training. A lot of current healthcare leaders were chosen to take on leadership because of their productivity, published research, solid clinical skills, or because they were great educators, Heifetz RA. Leadership Without Easy Answers; 1994 although they often do not have the skills to build a team, resulting in dysfunctional teams and having to deal with conflicts and chaos. The first steps of a Clinical Leadership Program is to gain insight in one’s personality, one’s personal skills and one’s leadership growth potential, because this gives information on one’s natural leadership style. The aim of our research is to gain insight in the personality traits of healthcare professionals who are leading teams and to check (a) whether Belgian physicians with leadership ambition, share certain preferences, (b) whether physicians differ from other healthcare staff in terms of personality, (c) whether our sample of Belgian physicians differs from a population of physicians in the United States of America.

**Methods:**

In-hospital physicians and non-physicians enrolled in a Clinical Leadership Program consented to participate. They explored their personal preferences across four dimensions, based on the Myers-Briggs Type Indicator (MBTI). Their most suitable MBTI profile was determined with a self-assessment and a complementary guidance of an MBTI-coach. Chi-squared tests and logistic regression were performed to check distributions across different MBTI-dimensions and to assess the relation with profession and location.

**Results:**

Among participating physicians significantly more preferences for ‘Thinking’ then for ‘Feeling’ were found. Non-physicians were found to be significantly more ‘Sensing’ and ‘Judging’ compared with physicians. No significant differences were found between physicians from our (Belgian) and the USA dataset.

**Conclusion:**

Preferences of physicians proved to be different from those of non-physicians. ‘ISTJ’ is the most frequent personality profile both in Belgian and USA physicians.

## Background

Organizations are forced to reinvent their business models because of the VUCA (Volatility, Uncertainty, Complexity, and Ambiguity) environment we are currently living in [1]. This is also true for healthcare (HC): multimorbidity, chronic diseases and the aging of the population, a shortage of health care providers, cost cuttings, the demand for high quality and innovations only adds to the challenges health care organizations are facing. To respond to these challenges, there is an emerging need for physician leaders to bring a fundamental change [2]. In this increasingly complex environment, organizations need to adapt to change quickly and appropriately in order to keep functioning. New evolutions develop continuously, and the urge for flexible leaders to guide these adjustments and guard the organization’s long-term objectives, arises. As stated by Johansson Hanse (2015): “a competent leader strives to bring out the best in his people, working towards a common goal”. Others defined it as servant leadership, wanting to bring out the best among the employees (e.g. nurses) and the community, and therefore servant leaders ordinarily have a good understanding of the importance of team members (physicians and nurses or health care professionals) in research and quality improvement activities [3, 4].

To become competent leaders, one has to be formally trained to tackle the challenges of today’s setting. Although a significant, positive relation between physicians’ leadership skills and patient outcomes, staff satisfaction and staff retention has been found two challenges to be tackled remain: physicians are not formally trained in clinical leadership skills during their physician training; additionally, a change in corporate culture in hospitals is needed [5]. Although physicians are overwhelmed with knowledge during medical school, they often are not introduced to leadership fundamentals. They are not familiar with business or corporate concepts, although they often enroll in team leading positions [6]. In order to face these deficits, doctors should be trained in leadership skills. The NHS developed an evidence-based behavioral model for Healthcare Leadership [7]. According to this model the following leadership skills are necessary in order to be a competent leader: creating a vision, inspiring shared purpose, leading with care, engaging the team, connecting their department, evaluating information, influencing for results, holding accountable and developing capability.

Research suggest that the enhancement of self-awareness leads to more effective leaders [8]. So the first step in leadership to reach your full potential: ‘Know Yourself’ [9]. Better leaders should be more proficient at assessing the level of their own behaviors and the impact that those behaviors have on others [10]. In accordance with this theory, leadership training programs should start with a self-assessment. Moreover, through MBTI, leaders get a chance to assess their preferences that impact their leadership styles [11, 12]. A recent constructed program, named ‘Clinical Leadership Program (CleP)’ starts its first training day with the module considering the theme ‘Me’ [13]. There are three levels for looking into yourself: personality, skills and values. The first level which determines one’s behavior is a person’s *innate* personality [14]. Another determinant of behavior is skills, which are *acquired* and can be trained, as opposed to personality. Furthermore, values are *culturally* defined and indicate standards of what is the socially acceptable [15]. Values can vary through time and between social classes. In an attempt to discover you’re innate personality, certain techniques can serve as useful tools,. since who you are often does not accord with who you want to be or how you want others to perceive you. In order to get a better understanding of your personality and skills, professional assessments such as the Myers-Briggs Type Indicator (MBTI), Neo-FFI or 360° questionnaires can be used. The two-former serve as self-reporting questionnaires, while the latter receives its input from multiple outside sources. However, there are a lot of other tools available which can be utilized for the same purpose; unraveling your personality and increasing self-awareness.

Considered sound and well-validated, the MBTI is the most widely used psychological instrument in the world [16]. It is used to explain the effects that personal preferences have on decision making and problem solving by looking at eight possible preferences people might have, based on two extremes for each of four different dimensions [17]. An individual’s profile results out of a combination of four out of the eight preferences. In total, sixteen profiles or ‘types’ are possible by composing those four preferences. The profiles are indicated by the use of colors according to their dominant trait: green, yellow, blue and red for ‘Sensing’ (S), ‘iNtuition’ (N), ‘Thinking’ (T) and ‘Feeling’ (F) respectively [10].

There is very little published research about the relationship between physicians, leaders and their personality types [18]. Healthcare Research states that there is an association between profession and MBTI type for managers, doctors and nurses [19]. Others have focused on the MBTI profiles of a specific medical specialty. A research paper by Stilwell et al. proved some aspects of personality to relate to one’s medical specialty choice. They claimed that those with a ‘Feeling’ preference chose Family Medicine significantly more often than those with ‘Thinking’ preference [20]. Of those selecting non-primary care, male, extraverted, and ‘Thinking’ types chose surgical specialties significantly more than women, introverted, and feeling types. Zardouz et al. identified ‘Introversion’, ‘Sensing’, ‘Thinking’ and ‘Judging’ as the most prevalent personality traits in prospective otolaryngology applicants [21]. Swanson et al. reported ‘Introversion’, ‘Sensing’, ‘Thinking’ and ‘Judging’ as the most common personality type in surgery residents [22]. Boyd and Brown identified ‘Extraversion’, ‘iNtuition’, ‘Thinking’ and ‘Judging’ to be the most common personality profiles of Emergency Department medical staff [23].

The aim of our research was to study MBTI preferences of Belgian (BE) healthcare professionals (physicians and other healthcare staff (HCS)) who are leading teams and finished a Clinical Leadership Program. The study allows us (a) to check whether Belgian physicians with leadership ambition share certain preferences, (b) whether physicians differ from other HC staff in terms of personality, and (c) whether our sample of Belgian physicians differs from a population of physicians in the United States of America (USA).

## Methods

### Population

In-hospital healthcare professionals who enrolled voluntarily in a Clinical Leadership Program (CLeP) participated in this research [13]. Participants were both physicians and non-physicians, the latter are referred to as HCS. CLeP consists of 5 days of training, covering a period of 5 months. The program was offered in two ways: either initiated by a hospital (Klina Brasschaat, OLVZ Aalst, ZNA Antwerp, UZA, AZ Heilige Familie & AZ Monica) or through self-enrollment in an Open Program. We distinguished middle managers from executives (top management) by using the following definition [24]: middle management is typically concerned with executing organizational plans which comply with the company’s policies. Usually, middle management controls a department and guides low-level managers towards better performance. Middle managers take care of effective group and intergroup functioning. Meanwhile, executives oversee the entire organization and carry great responsibilities. The development of strategic goals and plans as well as company policies are part of their tasks [25]. To compare the MBTI preferences of Belgian and USA physicians, a dataset from the MBTI® Form M U.S. national representative sample has been used as a benchmark. This is the most recently available MBTI data (2002–2006) obtained by CPP Inc.

### Procedure

Firstly, the participants got an introduction of leadership, personality and the use of the MBTI in a self-assessment. Next, the completion of the MBTI-questionnaire was performed in groups of maximum 20 participants, under the watching eye of the qualified MBTI-coach (physician with a certified MBTI-training). An initial MBTI-profile was acquired. An individual conversation with the coach and the group members complemented the initial result from the questionnaire. The results of both the questionnaire and the conversation were compared in order to find the most suitable MBTI profile. The participants received a booklet as well, containing additional information about their personality preferences. At the end of each session, all the participants had to write their profile on a big poster, displayed on the wall. Altogether, per group this process took half a day to be realized. All the participants were guided by the same MBTI-coach.

Because data were gathered from physicians and HCS (as opposed to patient surveys and/or medical records, this research was exempt from review-board approval under Belgian law. Prior to participation, physicians and HCS were explained the content of the research and the use of the data for scientific contributions. Participation was voluntarily and based on verbal consent. All participants agreed to store their data in our data in our database and for them is to be used in further research.

### MBTI assessment tool

**Fig. 1:**
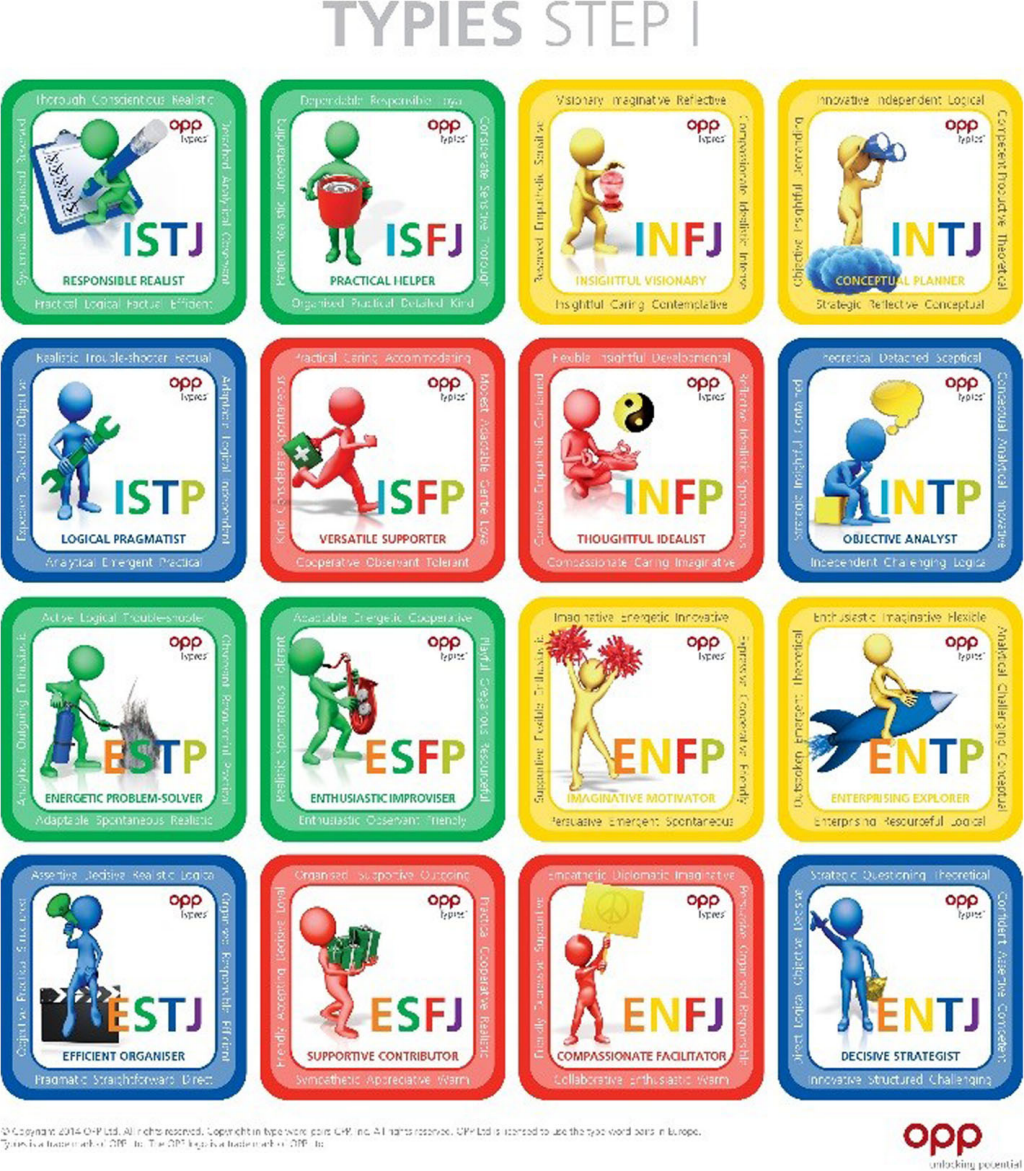
MBTI: Myers-Briggs Type Indicator (E: Extraversion; F: Feeling; I: Introversion; J: Judging; N: iNtuition; P: Perceiving; S: Sensing; T: Thinking)

One’s personality type represents someone’s preferences in four separate dimensions, with each category composed of two opposite poles. The four dichotomies describe key areas that combine to form the basis of a person’s personality. Each dichotomy can be briefly described as follows:. The first set of preferences determines where one gets one’s energy from: ‘Extraversion’ (E) or ‘Introversion’ (I). People with extravert preferences will tend to focus on the outside world for energy, where people with introvert preferences will focus on the inner world of ideas and experiences. The second set of preferences reveals whether people prefer to take information through ‘Sensing’ (S) or ‘iNtuition’ (N). People with a preference for sensing will prefer to take in information that is real and tangible, while people with a preference for iNtuition will prefer to focus on relationships and connections between facts. The third set of preferences determines whether one prefers to make decisions based on either ‘Thinking’ (T) or ‘Feeling’ (F) based on logic and objective principles or based on the concerns of the people involved respectively. The fourth set of preferences describes how people deal with the outer world; based on ‘Judging’ (J) or ‘Perceiving’ (P). ‘Judging’ means that one likes to live in a planned manner, while ‘Perceiving’ is more flexible. In each profile either ‘Sensing’, ‘iNtuition’, ‘Thinking’ or ‘Feeling’ is a dominant characteristic. The profiles are indicated with colors according to their dominant trait; green, yellow, blue and red and for ‘Sensing’, ‘iNtuition’, ‘Thinking’ and ‘Feeling’ respectively. All sixteen possible combinations are displayed in Fig. 1. Preferring one preference does not mean that the opposite preference is never used or cancelled out, rather it is about our natural way of doing things. This means that other preferences, that might seem less natural to us, can be developed as well.

### Statistical analysis

To analyze the MBTI preferences of Belgian physicians participating in CLeP, percentages per dimension were calculated and chi-squared tests were performed to check whether the preference for either category was significantly different from the 50% proportion, which represents the random preference scenario with a significance level of 0.05. Significant *p*-values indicate that physicians are not distributed randomly between the two levels of the category, and instead they have the tendency to score the level for which the percentage is higher. Secondly, we checked whether the profession (physician versus HCS) had an influence on the MBTI personality type distribution. More specifically, logistic regression was used to evaluate the influence of the profession on the category levels. Again, this was done separately for the different dimensions with a 0.05 significance level. Lastly, tests were performed to compare personality type distributions across databases (BE and USA physicians). Chi-squared tests are used to study the influence of the location (data set) on the MBTI personality type distribution only for physicians. This allows us to look at preferences per dimension for the two data sets. This comparison was carried out, assuming the two populations are comparable in terms of other aspects that could influence the MBTI personality type distribution.

## Results

### Population

Our sample consists of 180 healthcare professionals; 95 women and 85 men of eight different hospitals. 98 of them were hospital physicians and 82 HCS (head nurses, logistic managers, financial and IT-managers, administrative managers, …). Most of them were middle managers, responsible for a lower number of employees, often smaller teams or departments. Only 12 out of 98 physicians operated at executive level, managing entire organizations. Table 1 shows the institutions and affiliated participants with their location and the number of participants as well as their profession.

**Table 1:** Description of Sample Data

Institution	Number of participants	Profession
AZ Klina (*Brasschaat*)	22	Physicians and HCS
OLVZ (*Aalst*)	20	Physicians
ZNA (*Antwerp*)	51	Physicians
Antwerp University Hospital (UZA) (*Antwerp*)	55	HCS
AZ Heilige Familie (*Reet*)	11	HCS
AZ Monica (*Antwerp*)	4	HCS
CLeP Open Program (*Antwerp*)	17	Physicians

*HCS* = Healthcare staff

To compare Belgian physicians with USA physicians, MBTI data of 10,299 US physicians from the MBTI® national representative sample were used. This dataset is the largest available MBTI database (2002–2006) obtained by CPP Inc. and contains data from 5667 male and 4632 female specialists, among them are surgeons, allergists, dermatologists, anesthesiologists, internists, gynecologists, pediatricians, radiologists, urologists, neurologists, nuclear medicine physicians, ophthalmologists, pathologists and rehabilitation physicians. This distribution of hospital specializations is comparable with the Belgian dataset.

### MBTI preferences of Belgian physicians participating in CLeP

On the first dimension, 54% of the physicians show ‘Introversion’ as a preference over ‘Extraversion’ *(p-value 0.48).* On the second dimension, a slight majority of the physicians preferred ‘Sensing’ (54%) over ‘iNtuition’ (*(p-value 0.48)*. On the third dimension ‘Thinking’ vs ‘Feeling’, a significant difference was found *(p-value 0.03):* 61% of the physicians preferred ‘Thinking’, while the remaining preferred ‘Feeling’. Another difference was found concerning how physicians deal with the outer world: 60% are rather ‘Judging’ types, while 40% are rather ‘Perceiving’ types *(p-value 0.054)*.

### Comparing MBTI preferences of Belgian physicians and healthcare staff

**Fig. 2:**
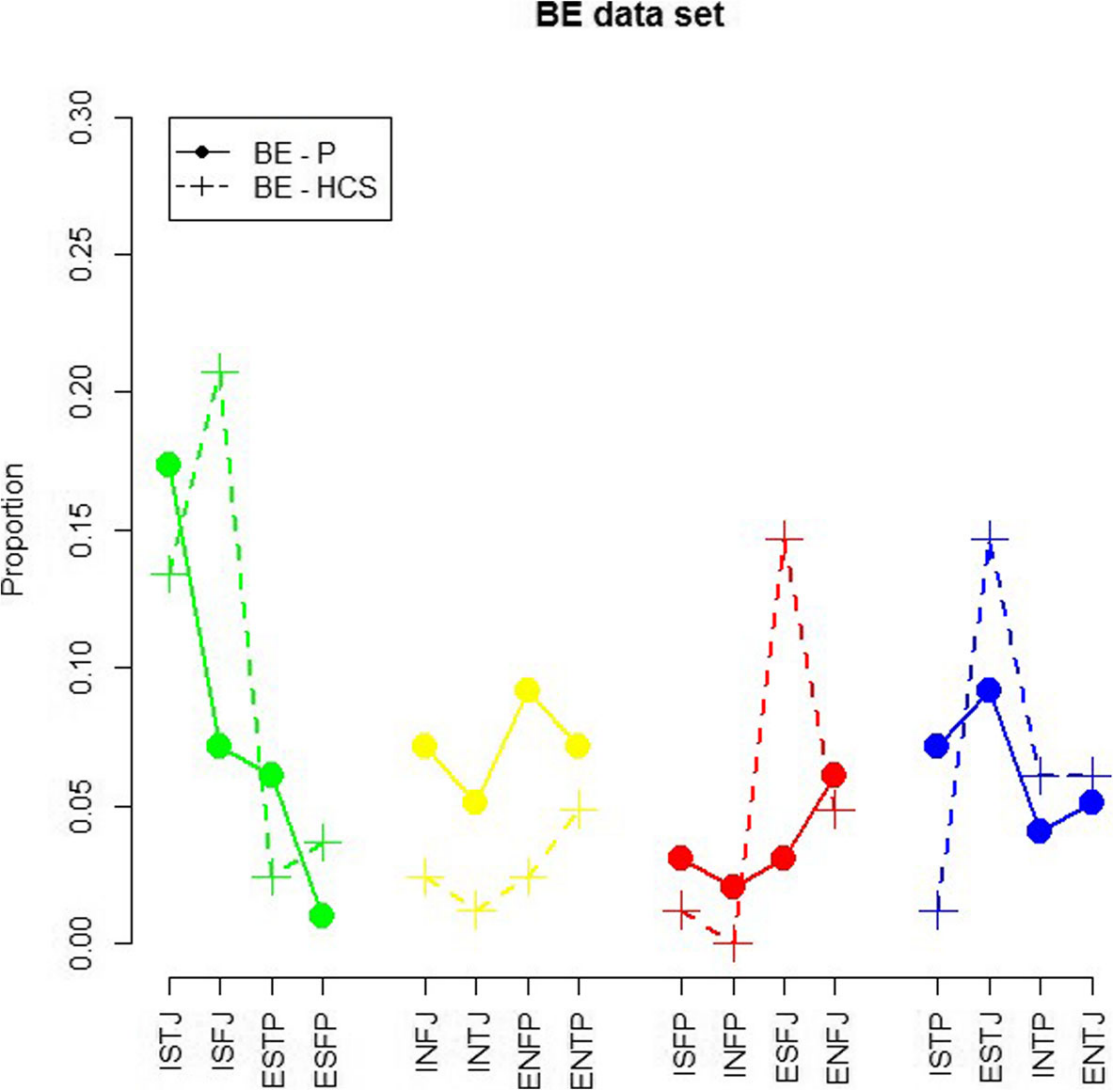
Distribution of personality types of Belgian Healthcare Professionals

At a first graphical exploratory data analysis, there seems to be differences in the distribution of MBTI personality types between physicians and other HCS. The personality types are grouped in four groups of four profiles, each group with a dominant characteristic: ‘S’-dominant in green, ‘N’-dominant in yellow, ‘F’-dominant in red, ‘T’-dominant in blue. Figure 2 shows the distribution of personality types of the Belgian sample for physicians and HCS. Some slight differences can be noticed. Considering the HCS, the most popular profiles are ‘ISFJ’, ‘ESFJ’ and ‘ESTJ’. When doing the same for physicians, we notice that ‘ISTJ’, ‘ENFP’ and ‘ESTJ’ are the most frequent.

Tests confirm that there is an association between profession and two dimensions, more specifically on the ‘Sensing’ vs ‘iNtuition’ dimension *(p-value 0.02)* and on the ‘Judging’ vs ‘Perceiving’ dimension *(p-value 0.02)* (Table 2). HCS have significantly different odds of ‘Sensing’ over ‘iNtuition’ (72% / 28%) compared with physicians (54% / 46%). On the fourth dimension, differences between professions are prevalent as well; HCS have significantly different odds of ‘Judging’ over ‘Perceiving’ (78% / 22%) compared with physicians (60% / 40%).

**Table 2:** Proportion by preference and profession in Belgian healthcare professionals

Dimension	Personality Trait	Physicians	HCS	p-value
1st dimension	Extroversion	46	50	0.838
	Introversion	49	51	
2nd dimension	iNtuition	46	28	0.026
	Sensing	54	72	
3rd dimension	Feeling	39	50	0.210
	Thinking	61	49	
4th dimension	Judging	60	78	0.020
	Perceiving	40	22	

### Comparing MBTI types of Belgian and US physicians

**Fig. 3:**
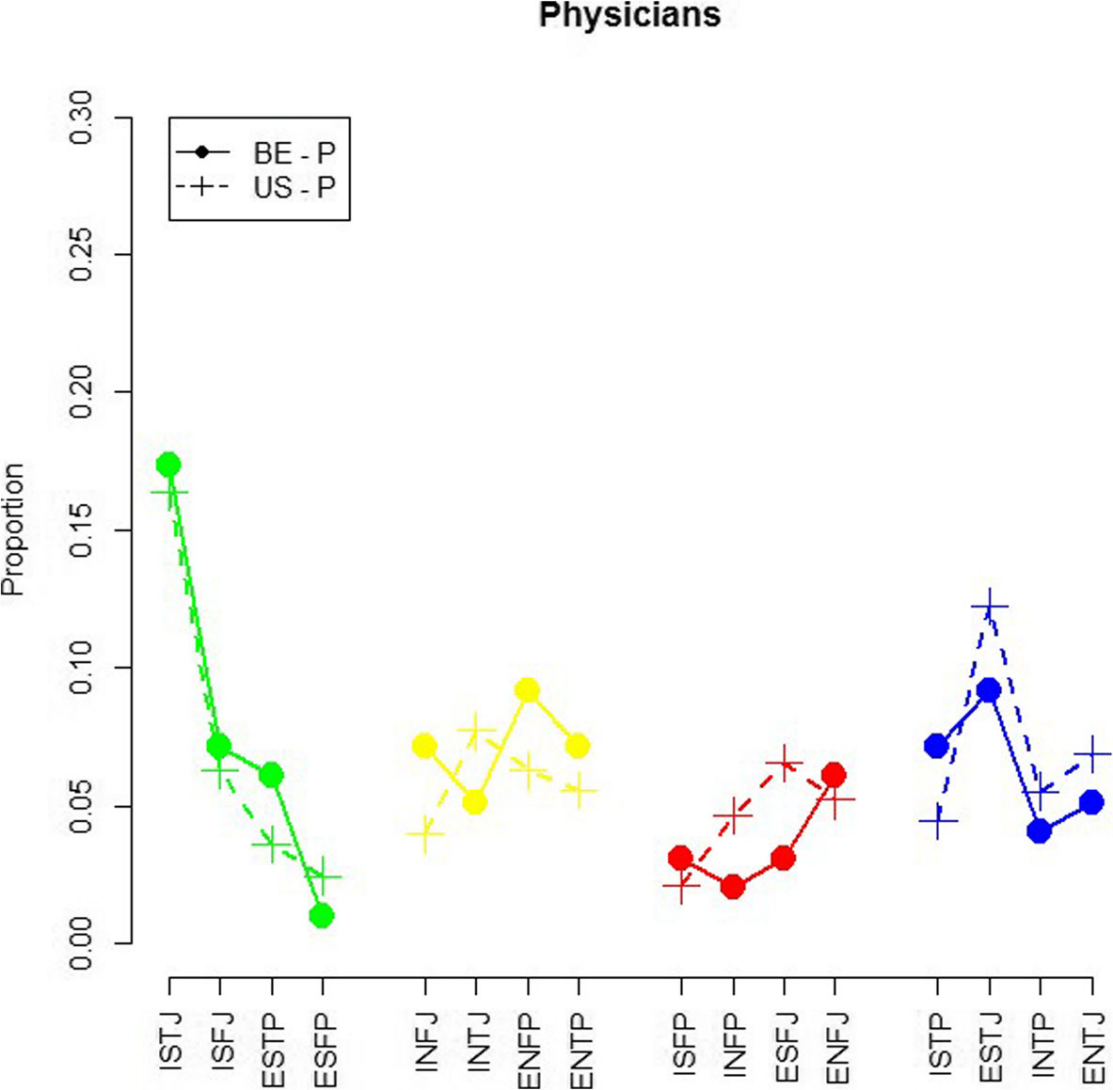
Distribution of personality types of Belgian and USA Physicians

Figure 3 shows the distribution of MBTI profiles of Belgian and USA physicians. Overall, some differences can be noticed, but the distributions seem to be rather comparable between the two data sets. Again, the personality types are put into four groups. Ranking profiles from most to least frequently occurring: ‘ISTJ’ is the most frequently occurring profile for the USA as well as for BE(respectively 16 and 17% for USA and BE physicians), followed by ‘ESTJ’ (12%) and ‘ENFP’ (9%) (USA vs BE) = and: ‘INTJ’ (8%) and ‘ENTP’/‘ISTJ’/‘INFJ’/‘ISFJ’ (7%) (USA vs BE). The least occurring profile in the USA data set is ‘ISFP’ (2%), while in Belgium it is ‘ESFP’ (1%). No significant association between location and personality type for physicians for all MBTI dimensions was found: chi-squared *p*-values were ‘EI’ = 0.63; ‘SN’ = 1.0; ‘TF’ = 0.88; ‘JP’ = 0.34. Even by distinguishing by gender, neither of the odds ratios were significantly different from 1.

## Discussion

A clinical leadership program which strives to develop the future generation of healthcare leaders, was modular constructed in five themes ‘Me’, ‘Team’, ‘Organization’, ‘Institution’ and ‘Society’. In the first module, personality and leadership skills were educated. All Belgian healthcare professionals in this program started with a MBTI personality test. The results of this MBTI test were studied. Within Belgian physicians, significantly more preferences for ‘Thinking’ than ‘Feeling’ were found. Additionally, more physicians were ‘Judging’ rather than ‘Perceiving’ types. Comparing the physicians with other HCS, there were significant differences: HCS proved to be more ‘Sensing’ and ‘Perceiving’. No significant differences were found comparing Belgian physicians with a large sample of USA physicians: the most frequent type for physicians was ‘ISTJ’: 17% in Belgium and 16% in the USA.

There are some limitations to this research. The first limitation is the limited number of participating Belgian healthcare professionals, which consequently results in small numbers per personality type. Another limitation is the testing itself. A self-assessment can still be influenced by the subjectivity of the participants. Furthermore, there are some remarks on the MBTI theory itself. It is not evident that the complex phenomenon of a personality can be captured exclusively in 16 types [19]. Since one’s personality is influenced by numerous factors such as experiences, relationships, values, it is far more complicated than capturing it ina four-letter acronym. Furthermore, it doesn’t mean that people with the same profiles are identical. There are nuances to what extent someone identifies with certain personality characteristics. Additionally, there is no convincing evidence to justify that knowing one’s type is a reliable or valid predictor of important behavioral condition [26]. Jung et al. claims that people do not change profiles during their lives, but it is possible to develop the different functions [27].

The sample consisted of several healthcare professionals of different hospitals across Flanders. Moreover, some of them participated autonomously and part of them enrolled from leadership development programs initiated by their hospitals. Although gender might have an influence, as stated by previous research, it was not studied in this research as it reduced the number of participants per group drastically [20].

The implementation of a MBTI personality test has several positive strengths as a first step in a clinical leadership program. First, withdrawal bias was limited because the whole procedure was coached by the same MBTI certified coach. Moreover, while refining the leadership program, it was that these leadership skills had to be instructed by a physician, trained and certified as a MBTI-mentor. Because it reduces the distance between mentor and leadership trainee. A physician MBTI-mentor is able to create a safe environment where trainees can discover their own and others’ personality and learn about team functioning and conflict management. This approach allows to blend pedagogic excellence with the experience and credibility that only physicians can offer [13]. Secondly, the procedure requires a self-assessment followed by a group process. At the end of the process, the different profiles of our participants were written on a flipchart in front of the class and participants gathered in teams. Relying on the theoretical background, participants were able to do introspection and learn about their behavior during the process. Participants were stimulated to reflect on the strengths of their own profile, but also on the strength of the other profiles. Thirdly, we strongly believe that a crucial, initial step in leadership development is introspection. ‘Knowing Yourself’ stimulates authentic leadership and it gives insights about our own behavior and its effect on the behavior of others [7]. For example, in conflicts, ‘Feeling’ and ‘Thinking’ preferences can clash because of their differences in communication style and decision process. A significant higher number of ‘Thinking’ in our hospital physicians was found and a trend of more ‘Feeling’ preference in our HCS. Because these types act totally different, it could explain why a lot of conflicts between hospital physicians and nurses arise. ‘Feeling’- types are behaviorally inclined to verbalize their thoughts and feelings, they take their decision by stepping in the situations and taking into account the feelings of their team members. ‘Thinking’- types could neglect their own and others’ feelings. They take decisions on objective data and by stepping out of the situation. This creates conflicts because they do not understand each other or they do not respect the different approach of the other preference. A relationship featuring two individuals who share the same MBTI preference is often more manageable, even if they share conflicting views [28].

There is no ‘superior profile’ [29]. It is crucial to recognize particular strengths and weaknesses inherent to each profile. Specific profiles interact in specific ways, using different dynamics. Getting insights in these dynamics can be of good use when considering teams: it is preferable to have a good mix of different types in a team [30]. One necessary condition is having respect for different personalities and their particular behavior. The most frequent type in physicians is ‘ISTJ’. ISTJ’s are responsible organizers, driven to create and enforce order within systems and institutions. They tend to have a procedure for everything they do. Reliable and conscientiously, ISTJ’s want to uphold tradition and follow regulations [31]. ISTJ’s like to know what the rules of the game, valuing predictability more than imagination. ISTJ’s are hardworking and will persist until a task is done. They are logical and methodical, and often enjoy tasks that require them to use step-by-step reasoning to solve a problem. They are meticulous in their attention to details, and examine things closely to be sure they are correct [31]. However, ISTJ’s are rather conservative. This means that they have less preference for change. This type will be challenged in a VUCA-world were change will be the new normal. This could be the explanation why it is often difficult to implement change in hospitals.

Although a higher prevalence of ‘TJ’ combination is found in physicians following a leadership program, it is not a prerequisite for successful leadership [18]. For physicians with a preference for ‘Feeling’ or ‘Perceiving’ to function well, awareness of their ‘opposites’ is needed.

## Conclusion

To conclude, physicians who have interest in following a leadership development program do not substantially differ from the general population of physicians. Differences can be noticed within the group of physicians and between nurses and physicians. This research contributes to insights into the personality of Belgian physicians and HCS which can be of good use when considering teams when pursuing flourishing organizations.

## Abbreviations

BE: Belgian/Belgium
CLeP: Clinical Leadership Program
E: Extraversion
F: Feeling
HCS: Healthcare staff
I: Introversion
J: Judging
MBTI: Myers-Briggs type indicator
N: iNtuition
P: Perceiving
S: Sensing
T: Thinking
USA: United States of America
VUCA: Volatility, Uncertainty, Complexity, and Ambiguity

## References

[1] Sturmberg JP (2013). Martin CM. Handbook of systems and complexity in health: chapter 1.

[2] Mountford J, Webb C. When clinicians lead. McKinsey QHealthcare. 2009

[3] West M, Armit K, Loewenthal L, Eckert R, West T, Lee A. Leadership and Leadership Development in Health Care : The Evidence Base Contents. In: FMLM, Center for Creative Leadership, The King’s Fund. London: Faculty of Medical Leadership and Management with The King’s Fund and the Center for Creative Leadership; 2015.

[4] West M, Lyubrovnikova J (2013). Illusions of team working in health care. J Health Organ Manag.

[5] van der Wal MA, Scheele F, Schönrock-Adema J, Jaarsma ADC, Cohen-Schotanus J. Leadership in the clinical workplace: what residents report to observe and supervisors report to display: an exploratory questionnaire study. BMC Med Educ. 2015;15(1). 10.1186/s12909-015-0480-5. 10.1186/s12909-015-0480-5 PMC4630964 26525409

[6] Heifetz RA. Leadership without easy answers. Cambridge: Harvard University Press; 1994.

[7] Storey J, Holti R (2013). Towards a New Model of Leadership for the NHS.

[8] Van Velsor E, Taylor S, Leslie J (1993). An examination of the relationships among self-perception accuracy, self-awareness, gender, and leader effectiveness. Hum Resour Manag.

[9] Peterson DB, Hicks MD (1995). NoDevelopment first: strategies for self-development.

[10] McCarthy AM, Garavan TN (1999). Developing self-awareness in the managerial career development process: the value of 360-degree feedback and the MBTI. J Eur Ind Train.

[11] Watland KH (2009). The Myers-Briggs type Indicator as a tool for leadership development in management education programs: What’s type got to do with it?. Transform Dialogues Teach Learn J.

[12] Sethuraman K, Suresh J (2014). Effective leadership styles. Int Bus Res.

[13] Claes N, Brabanders V. Leadership Training Program for Medical Staff in Belgium. 2016;5(4):281–287. doi:10.5430/ijhe.v5n4p281.

[14] Griffiths P (2017). The Disctinction Between Innate and Acquired Characteristics. In: The Stanf Encycl Philos Spring.

[15] Lapinski MK, Rimal RN (2005). An explication of social norms. Commun Theory.

[16] Coan R. The Myers-Briggs type indicator. In: In OK Buros, ed. The Eight Mental Measurements Handbook. Highland Park, NY: Gryphon; 1978:970–975.

[17] Keirsey D, Bates M (1984). Please Understand Me*:* Character and temperament types. B&D Books.

[18] Aranda R, Tilton S (2013). Myers-Briggs personality preferences may enhance physician leadership success in non-clinical jobs. Physician Exec.

[19] Pittenger David J. (1993). The Utility of the Myers-Briggs Type Indicator. Review of Educational Research.

[20] Stilwell NA, Wallick MM, Thal SE, Burleson JA (2000). Myers-Briggs type and medical specialty choice: a new look at an old question. Teachine Learn Med.

[21] Zardouz S, German MA, Wu EC, Djalilian HR (2011). Personality types of otolaryngology resident applicants as described by the Myers-Briggs type Indicator. Otolaryngol Neck Surg.

[22] Swanson JA, Antonoff MB, D’Cunha J, Maddaus MA (2010). Personality profiling of the modern surgical trainee: insights into generation X. J Surg Educ.

[23] Boyd Russell, Brown Terry (2005). Pilot study of Myers Briggs Type Indicator personality profiling in emergency department senior medical staff. Emergency Medicine Australasia.

[24] Boundless. Mintzberg’s Management Roles. Boundless Management. 1990.

[25] Business B. Management Levels: A Hierarchical View.; 2016.

[26] Hansen D, Jacobs N, Bex S, D’Haene G, Dendale P, Claes N (2011). Are fixed-rate step tests medically safe for assessing physical fitness?. Eur J Appl Physiol.

[27] Taylor F, Jung CG. In: Papadopoulos PK, editor. The Structure and Dynamics of the Psyche (Vol. 2). London: Taylor & Francis Ltd; 1992.

[28] ABC Consulting. Myers-Briggs type Indicator step I communication style report. Sunnyvale: CPP; 2016.

[29] Cunningham L. "Myers-Briggs: Does is pay to know your type?". The Washington Post. 2012.

[30] Shorr K. Using the Myers-Briggs type Indicator to improve teamwork. Hum Resour B2B insights. 2012.

[31] Myers, Briggs. MBTI Manual. 3rd ed. Sunnyvale: CPP; 2001.

